# Lessons about physiological relevance learned from large-scale meta-analysis of co-expression networks in brain organoids

**DOI:** 10.1371/journal.pbio.3002965

**Published:** 2024-12-18

**Authors:** Yoshiaki Tanaka

**Affiliations:** Maisonneuve-Rosemont Hospital Research Centre (CRHMR), Department of Medicine, University of Montreal, Montreal, Quebec, Canada

## Abstract

Integrative analysis of publicly available scRNA-seq data facilitates deeper understanding of biological phenomena with strong statistical power and high resolution. A new study in this issue of *PLOS Biology* examined the fidelity of various brain organoid protocols in reference to human primary developing brain by gene co-expression relationships with million-scale collection of public scRNA-seq data sets.

## Abstract 2

Despite the recent rapid advances in neural organoid culture systems, it is unclear how closely they mimic primary human tissue physiology. This Primer explores a new study in PLOS Biology that conducts a meta-analysis of single-cell sequencing data to to examine the fidelity of neural organoid protocols.

Brain organoids are in vitro-cultured three-dimensional (3D) aggregates and intrigue models that allow us to deeply investigate obscure early human brain development and human-specific characteristics of neurological disorders. Over the past few years, scientific communities have endeavored to establish protocols that generate brain organoids representing whole brain or specific brain areas, including cortex, midbrain, thalamus, hypothalamus, medial ganglionic eminence, choroid plexus, brain stem, and cerebellum [1]. In addition, non-ectodermal cell types, such as microglia and vascular endothelial cells, which usually cannot be differentiated by the conventional protocols, were also successfully introduced into the brain organoids by transgene or co-culture methods [1–3]. Despite the recent rapid advance of the 3D culture systems, it is still a “red-hot” topic how closely the brain organoids mimic primary human tissue physiologies.

Since the brain organoid is composed of multiple cell types, single-cell transcriptome profiling has been often employed to address the composition of the cell types and the molecular characterization of each cell in the brain organoids. Increasing number of the single-cell transcriptomics data in public repositories, such as NCBI Gene Expression Omnibus (GEO), gives rise to various secondary synthetic analyses that address the protocol-to-protocol variabilities and resemblance and distinctness of the organoids with primary human brains. Early studies have employed hundreds of thousands number of cells from the brain organoids and human fetal brain samples and demonstrated elevation of cellular stresses, experimental validations, specification of regional identities in the brain organoids [4–7].

A newly published meta-study led by Werner and Gillis demonstrated the preservation of co-expression networks between primary developing human brains and the brain organoids [8]. Since genes involved in the related biological processes are likely to be simultaneously expressed, the co-expression is one of reliable measurements to assess functional similarity between 2 different systems. Notably, they encompassed gene expression profiles of almost 3 millions of cells (9 publications) from multiple primary developing brain regions and 1.6 millions of cells (25 publications) from different brain organoid protocols. The huge size of scRNA-seq data collection contributes to not only statistic power, but also facilitates to address the robustness and the variability across data sets.

To analyze the co-expression network, they employed MetaMarkers that are robust gene sets in each cell type and independent from temporal and regional variations of brains [9]. Using gene expression profiles from primary human developing brains, they identified primary tissue MetaMarkers in 6 different cell type: glutamatergic neuron, GABAergic neuron, dividing progenitor, neuronal progenitor, intermediate progenitor, and non-neuronal cell. These MetaMarker gene sets were subsequently used to evaluate whether they are identical between primary developing brains and the organoids. Although high variability of MetaMarker co-expression was observed in individual organoid data sets, the co-expression network was comparable with primary developing brains when all data sets were aggregated. However, the preservation of the primary developing brain co-expression was more variable across individual organoid data sets in most of cell types. Furthermore, the co-expression network from adult brains, which were composed of hundreds of thousands cells, was not perfectly captured in glutamatergic and GABAergic neurons of the organoid, since the organoids more closely recapitulate co-expression in fetal brain than adult.

Their co-expression analyses also reported new aspects of the brain organoids. For example, elevation of cell stress-related genes, such as endoplasmic reticulum (ER), glycolysis, and oxidative stress, in the brain organoid also has been reported in previous integrative analyses [4,5]. However, it is still controversial if the cell stress-related genes disrupt cell type identities. This study demonstrated weak negative association of the cell type-specific preserved co-expression with ER gene expression and no association with gene expression involved in glycolysis and oxidative stress. In addition, the expression of stress-related genes was highly variable across the organoid data sets. Although the inherent cellular stress is one of common issues that should be solved for the primary brain fidelity, their effect on cell identifies in the brain organoids seem to be minimal with regard to the co-expression network.

Recently, Human Cell Atlas Organoid Biological Network also published a comprehensive collection of single-cell transcriptomics of the brain organoids, termed human neural organoid cell atlas (HNOCA), that covers about 1.7 millions of cells (26 publications and 2 unpublished works) from different protocols [10]. Only data sets from 10 publications were shared with Werner and Gillis’s data collections, maybe due to differences of collection and curation rules (Fig 1). However, both studies purposed similar objective how transcriptomics of in vitro-generated organoid cell types resemble across protocols and to those in primary developing brains. While Werner and Gillis used the co-expression network, the Human Cell Atlas employed Bayesian inference and deep learning to integrate the organoid cells into the primary reference atlas. Despite the different analytic strategies, both studies have the same conclusion that the cell stress was elevated as universal characteristics in the organoids, but have trivial effects on the cell identities. Overall, these parallel efforts by different groups benchmarked the fidelity of primary developing brains in individual organoids and provide essential insights for future translational applications in disease modeling and study of neurodevelopmental processes.

**Fig 1:**
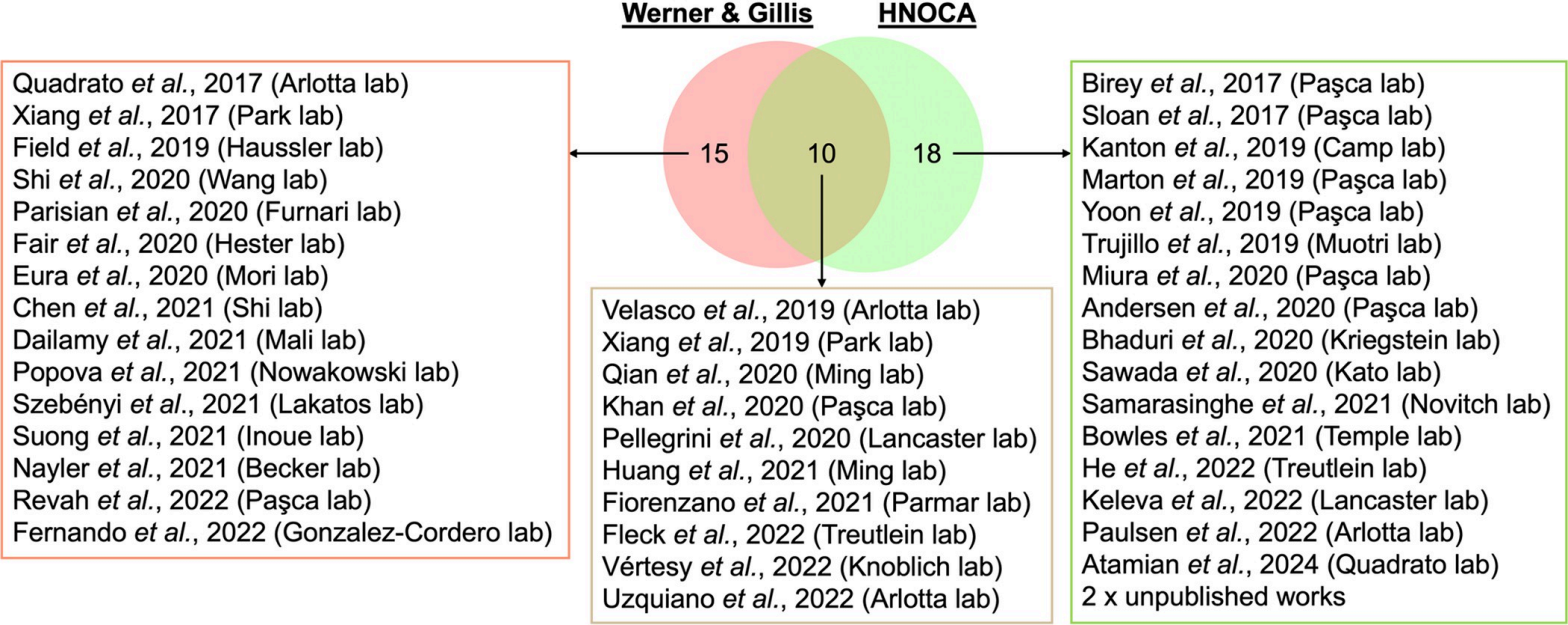
Comparison of single-cell transcriptomic collection between Werner and Gillis study and HNOCA. Common and unique publications to each comprehensive study are shown.

Collectively, Werner and Gillis proposed the co-expression network as one of cell type-specific measurement that can assess the primary brain fidelity and the reproducibility of authentic molecular identities in 3D in vitro culture. The growing secondary analyses of single-cell transcriptome data have clarified potential gaps in the organoids and accelerate rationalization of protocol procedures and medium composition for further compatibility with primary brains.
